# Clinical significance of age at diagnosis among young non-small cell lung cancer patients under 40 years old: a population-based study

**DOI:** 10.18632/oncotarget.5524

**Published:** 2015-10-26

**Authors:** Mina Liu, Xuwei Cai, Wen Yu, Changxing Lv, Xiaolong Fu

**Affiliations:** ^1^ Department of Radiation Oncology, Shanghai Chest Hospital, Shanghai Jiao Tong University, Shanghai, China

**Keywords:** NSCLC, age, SEER

## Abstract

**Background:**

Young non-small cell lung cancer (NSCLC) patients under the age of 40 can further be categorized into different age subgroups. Whether they have homogeneous clinical features and survival outcomes remains unexplored.

**Methods:**

Information of 4623 NSCLC patients up to 40 years old from 1988 to 2012 was retrieved from the Surveillance, Epidemiology, and End Results (SEER) database. Clinicopathologic characteristics and survival outcomes were compared between patients diagnosed at 18–30 years old (younger group) and those at 31–40 years old (older group).

**Results:**

The proportion of patients in the younger group among all lung cancer patients was stable between 1988 and 2012. However, the proportion of patients in the older group decreased from 1.2% to 0.5%. The younger patients had a higher proportion of adenocarcinoma (*P* = 0.016), a lower proportion of large cell carcinoma (*P* = 0.008), a higher proportion of stage I disease (*P* = 0.002) and a lower proportion of stage III disease (*P* < 0.001). The younger patients had significantly better lung cancer-specific survival (LCSS) in the whole cohort (*P* < 0.001) and in the subgroup of patients with stage I (*P* = 0.038) or stage IV (*P* < 0.001) disease. Multivariate survival analysis showed that patients under 30 years old was an independent predictor of both better LCSS (*P* = 0.010) and overall survival (OS) (*P* = 0.018).

**Conclusions:**

Adult NSCLC patients under 30 years old had distinctive clinicopathologic characteristics and survival outcomes compared to patients diagnosed at 31–40 years old.

## INTRODUCTION

According to the recently released global cancer statistics [1], lung cancer is the leading cause of death from malignancy among males in both more and less developed countries, and the leading cause of cancer death among females in more developed countries. Non-small cell lung cancer (NSCLC) in young adults (≤ 40 years old) accounts for a very small proportion as this disease usually occurs in people at older age. Several population-based studies have compared the characteristics between young and old patients with NSCLC using age thresholds ranging from 40 to 50 [2–8]. Young patients with lung cancer were more likely to be females and have adenocarcinoma histology, while the prognosis of young lung cancer patients compared to the older counterparts was controversial [2–8]. However, NSCLC patients under the age of 40 can further be categorized into different age subgroups (for example, 18–30, 31–40). Whether they have homogeneous clinical features and survival outcomes remains unexplored. This study aimed to compare clinicopathologic characteristics and survival outcomes in age subgroups of young NSCLC patients (below 40 years) using the Surveillance, Epidemiology, and End Results (SEER) database.

## RESULTS

**Figure 1:**
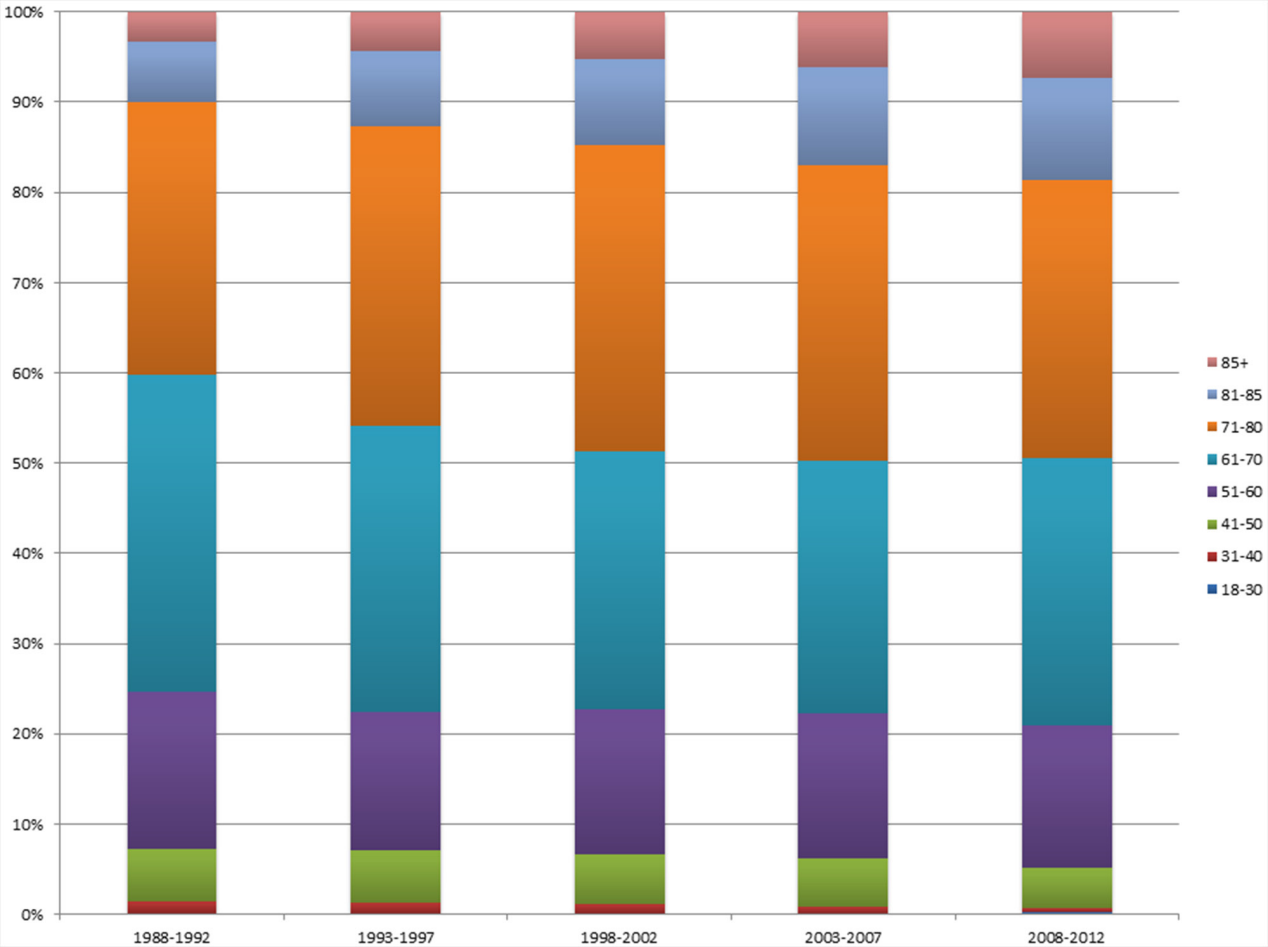
The proportion of lung cancer patients in age groups “18–30”, “31–40”, “41–50”, “51–60”, “61–70”, “71–80”, “81–85” and “85+” from 1988 to 2012.

**Figure 2:**
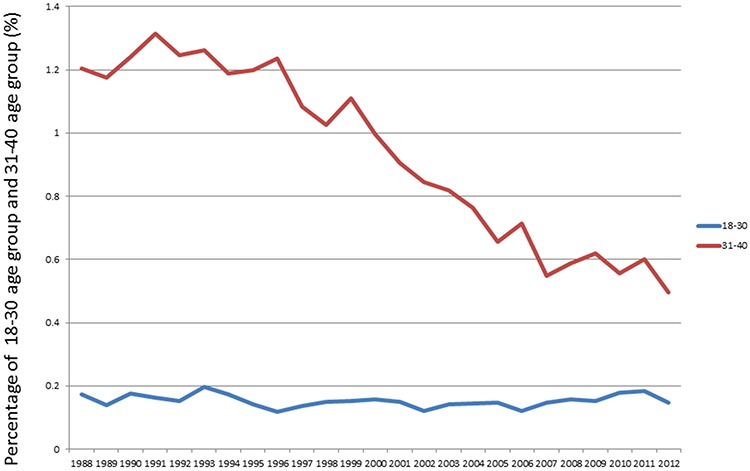
The proportions of patients in the two younger age groups of “18–30” and “31–40” among all lung cancer patients over time.

The proportion of lung cancer patients in age groups “18–30”, “31–40”, “41–50”, “51–60”, “61–70”, “71–80”, “81–85” and “85+” from 1988 to 2012 were demonstrated in Figure 1. The proportions of patients in the two younger age groups of “18–30” and “31–40” among all lung cancer patients over time were listed in Figure 2. The percentage of patients in the “18–30” age group was stable over the years, ranging from 0.12% to 0.20%. However, the proportion of patients in the “31–40” age group increased from 1.2% in 1988 to 1.3% in 1991, then decreased gradually to 0.5% in 2012.

A total of 4623 young NSCLC patients (up to 40 years old) were identified from 1988 to 2012, of which 429 were 18–30 years (the younger group), and 4194 were 31–40 years (the older group) (Table 1). The median age at diagnosis was 28 and 38 years in the younger group and the older group, respectively.

**Table 1:** Clinicopathologic characteristics of young non-small cell lung cancer patients under 40 years old according to age at diagnosis (18–30 and 31–40)

Variables	The “18–30” age group	The “31–40” age group	*P*
Gender			0.554
Female	211 (49.2%)	2000 (47.7%)	
Male	218 (50.8%)	2194 (52.3%)	
Race			
White	301 (70.2%)	2902 (69.2%)	0.679
African American	55 (12.8%)	753 (18.0%)	0.008
Others	73 (17.0%)	539 (12.9%)	0.015
Year of diagnosis			<0.001
1988–1999	85 (19.8%)	1521 (36.3%)	
2000–2012	344 (80.2%)	2673 (63.7%)	
Histology			
Adenocarcinoma	284 (66.2%)	2527 (60.3%)	0.016
Squamous	49 (11.4%)	457 (10.9%)	0.740
Large cell	20 (4.7%)	347 (8.3%)	0.008
Adenosquamous	10 (2.3%)	76 (1.8%)	0.449
NOS	66 (15.4%)	764 (18.2%)	0.145
Undifferentiated	0 (0)	23 (0.5%)	0.265
Stage			
I	67 (15.6%)	446 (10.6%)	0.002
II	14 (3.3%)	144 (3.4%)	0.853
III	75 (17.5%)	1080 (25.8%)	<0.001
IV	273 (63.6%)	2524 (60.2%)	0.163
Surgery			0.004
Yes	156 (36.4%)	1205 (28.7%)	
No	270 (62.9%)	2957 (70.5%)	
Unknown	3 (0.7%)	32 (0.8%)	
Radiation			<0.001
Yes	179 (41.7%)	2358 (56.2%)	
No	246 (57.3%)	1743 (41.6%)	
Unknown	4 (0.9%)	93 (2.2%)	
Lymph nodes removed			0.022
0	261 (60.8%)	2783(66.4%)	
1–5*	62 (14.5%)	542(12.9%)	
No less than 6	67 (15.6%)	464 (11.1%)	
Unknown	39 (9.1%)	405 (9.6%)	
Lymph nodes positive			0.033
0	76 (17.7%)	570 (13.6%)	
1*	32 (7.5%)	315 (7.5%)	
No less than 2	30 (7.0%)	220 (5.2%)	
Unknown	291 (67.8%)	3089(73.7%)	
Tumor Grade			<0.001
Well differentiated	43 (10.0%)	169 (4.0%)	<0.001
Moderately differentiated	55 (12.8%)	560 (13.4%)	0.823
Poorly differentiated	124 (28.9%)	1498 (35.7%)	0.005
Undifferentiated; anaplastic	24 (5.6%)	257 (6.1%)	0.750
Unknown	183 (42.7%)	1710 (40.8%)	0.471
Second cancer deaths	31 (7.2%)	250 (6.0%)	0.289

Abbreviations: NOS, not otherwise specified

Note: *The median number of lymph nodes removed was 5. The median number of lymph nodes positive was 1.

A higher proportion of the younger group patients were diagnosed after 2000 (80.2% vs. 63.7%, *P* < 0.001). Gender distribution was comparable in the two age subgroups. There were a significantly higher proportion of African American in the older group compared to the younger group (18.0% vs. 12.8%, *P* = 0.008).

Adenocarcinoma was the predominant histology in both groups. However, patients in the younger group had a higher proportion of this histology (66.2% vs. 60.3%, *P* = 0.016). Large cell carcinoma was more common in the older group compared to the younger group (8.3% vs. 4.7%, *P* = 0.008). The proportions of squamous cell carcinoma and adenosquamous cell carcinoma were comparable between the two groups. The younger group had a significantly higher proportion of well differentiated tumors (10.0% vs. 4.0%, *P* < 0.001), whereas the older group had a significantly higher proportion of poorly differentiated tumors (6.1% vs. 5.6%, *P* = 0.005).

Stage distribution was different in the two groups. The younger group had a higher proportion of stage I disease (15.6% vs. 10.6%, *P* = 0.002), whereas the older group had a higher proportion of stage III disease (25.8% vs. 17.5%, *P* < 0.001). Comparable proportions of patients in the two groups were diagnosed as stage II and stage IV.

SEER database provided information of surgery and radiation. A higher proportion of patients in the younger group received surgery (36.4% vs. 28.7%, *P* = 0.004), whereas the proportion of patients undergoing radiation therapy was significantly higher in the older group (56.2% vs. 41.7%, *P* < 0.001). There was a higher proportion of patients in the younger group receiving lymph nodes resections (30.1% vs 24%, *P* = 0.007). A significantly higher number of positive lymph nodes was found in the younger group (*P* = 0.033).

### Survival analysis

**Figure 3:**
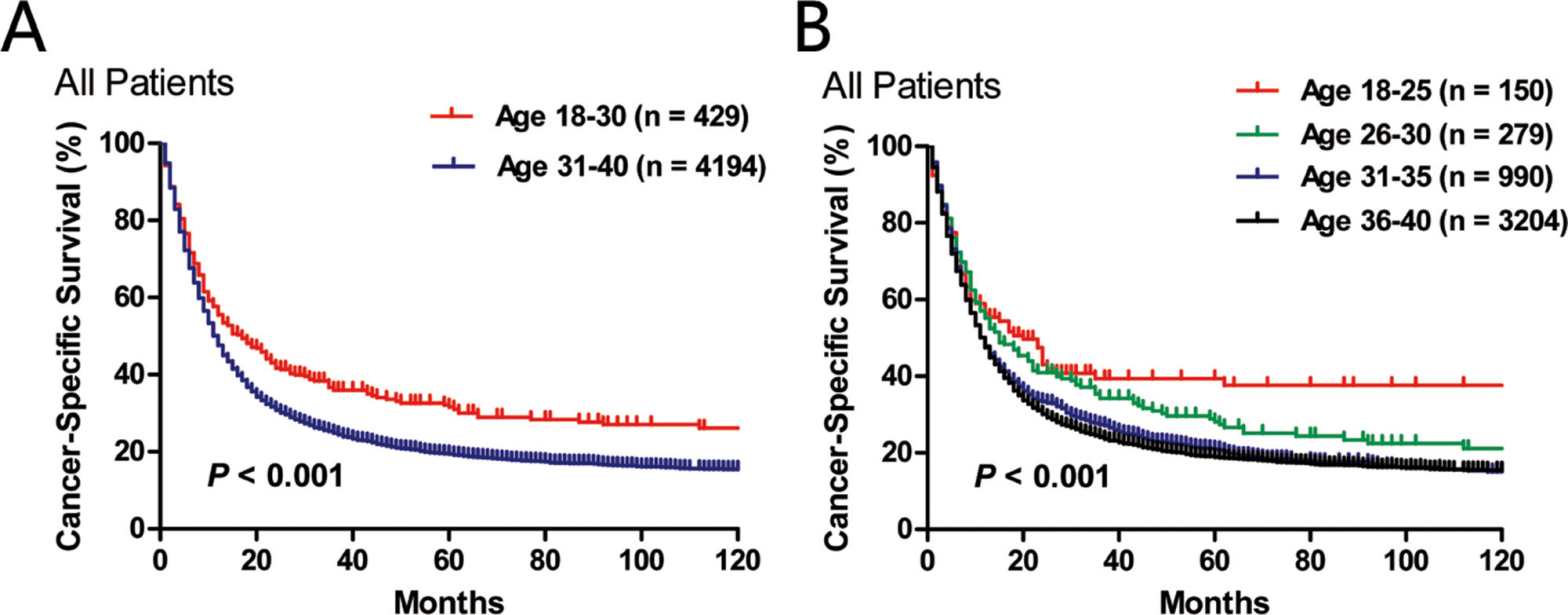
Comparison of lung cancer-specific survival in two A. or four B. age subgroups.

First, we compared the lung cancer-specific survival between the “18–30” and the “31–40” age subgroups (Figure 3A), and found that patients in the younger group had significantly higher LCSS (*P* < 0.001). We further categorized patients into four age subgroups: 18–25, 26–30, 31–35 and 36–40 (Figure 3B). There was a trend towards decreased LCSS as age increased (*P* < 0.001).

**Figure 4:**
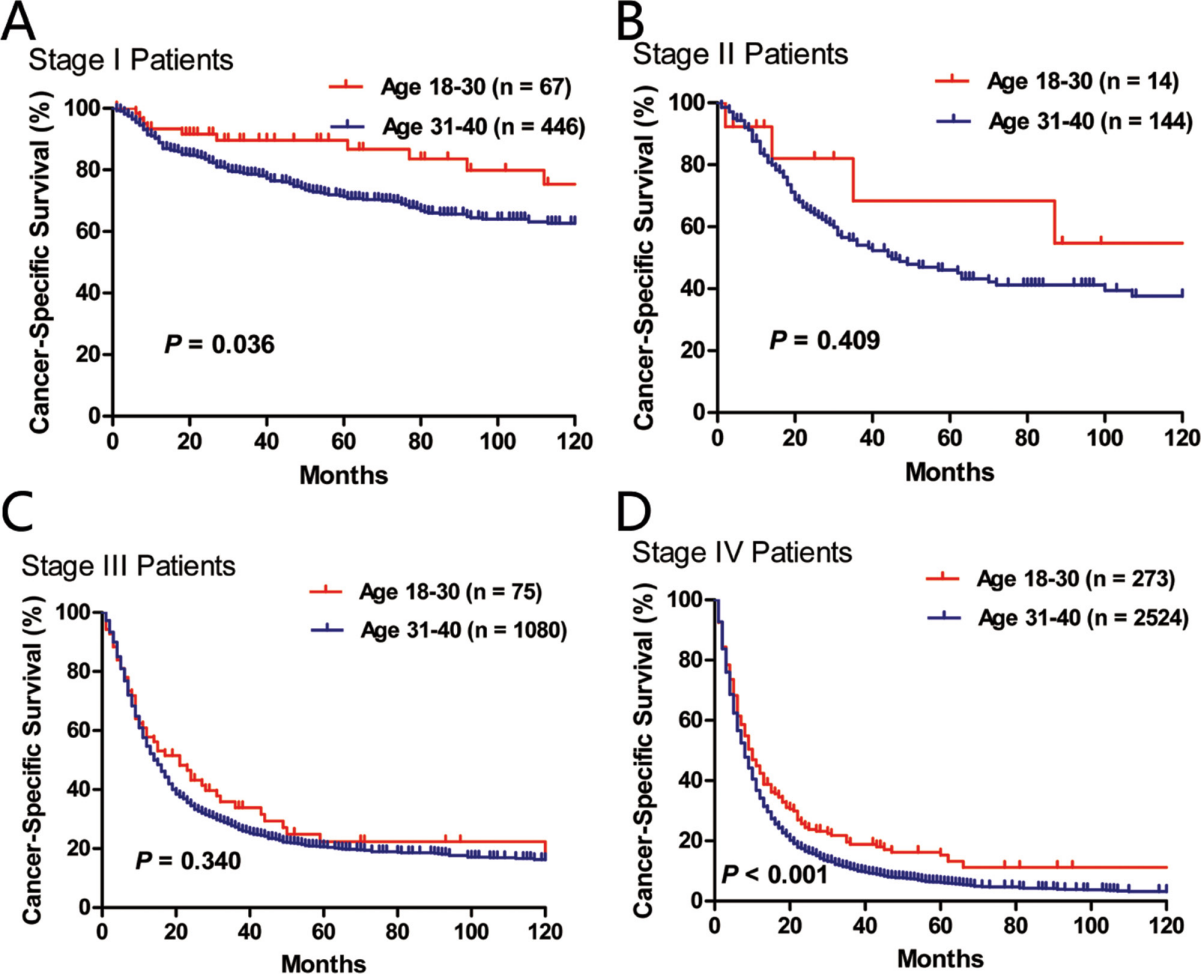
Comparison of cancer-specific survival between age subgroups (18–30 vs. 31–40) in stage I A., stage II B., stage III C. and stage IV D. patients.

When we limited the survival comparisons between the “18–30” and the “31–40” age subgroups in stage I, stage II, stage III or stage IV patients, we found that the younger patients had significantly better LCSS in stage I (*P* = 0.038) and stage IV (*P* < 0.001) groups. There was a trend towards higher LCSS for the younger group with stage II or stage III disease, although statistical significance was not achieved (Figure 4).

Multivariate survival analysis adjusted for race, sex, year of diagnosis, histology, tumor grade, AJCC stage, surgery and radiotherapy showed that patients under 30 years old was an independent predictor of both better LCSS (HR = 0.846, 95% CI: 0.745–0.961, *P* = 0.010) and better OS (HR = 0.864, 95% CI: 0.765–0.976, *P* = 0.018). We further performed multivariate survival analysis adjusted for race, sex, year of diagnosis, histology, tumor grade, AJCC stage, radiotherapy, lymph nodes removed and lymph nodes positive in patients undergoing surgery. Younger age at diagnosis was not an independent predictor of LCSS (HR = 0.807, 95% CI: 0.627–1.039, *P* = 0.097) or better OS (HR = 0.824 95% CI: 0.648–1.046, *P* = 0.112). Moreover, in patients who had not received surgery, younger age was also not a significant prognostic factor (LCSS: HR = 0.876, 95% CI: 0.755–1.016, *P* = 0.080; OS: HR = 0.899, 95% CI: 0.780–1.037, *P* = 0.144) in multivariate analysis adjusted for race, sex, year of diagnosis, histology, tumor grade, AJCC stage and radiotherapy.

A total of 281 (6.1%) young NSCLC patients died of second cancers other than lung cancer. Among them, 31 patients (7.2%) were in the younger group and 250 patients (6.0%) were in the older group (*P* = 0.289).

## DISCUSSION

Several population-based studies have investigated the clinical features and prognosis of young NSCLC in comparison to their older counterparts. The cutoff age to denote a young lung cancer patient ranged from 40 to 50 years. However, young age also contained wide age range of population, which suggested that different age subgroups of young NSCLC might have distinctive clinical characteristics and survival outcomes. Therefore, we compared the clinicopathologic characteristics and survival outcomes in age subgroups of young NSCLC patients under 40 years old in this study.

Using SEER database, Anish Thomas and colleagues found that the incidence of NSCLC in the young (< 40 years old) decreased from 1978 to 2010 [9]. Our data further showed that the percentage of patients in the “18–30” age group was stable over the years. However, the proportion of patients in the “31–40” age group decreased over time. A previous study reported that lung cancer death rates in young adults aged between 30 and 39 years correlated strongly and inversely with the individual state tobacco control efforts [10]. Better tobacco control is one of the possible reasons for the decreasing incidence of lung cancer in patients 31–40 years old. Lung cancer in patients under 30 may be more closely correlated to biological factors, although these information were not available in SEER database.

We observed a higher proportion of “18–30” years-old patients were diagnosed after 2000 compared to the “31–40” years-old group (80.2% vs. 63.7%, *P* < 0.001). Gender distribution was not significantly different in the two age subgroups, although previous studies found a higher proportion of female patients in the young NSCLC [4, 6, 7]. In a previous report using SEER database, Subramanian and colleagues [4] found that African Americans were more prevalent in NSCLC patients younger than 40 compared to those who were older than 40 and suggested that lung cancer might affect African Americans at an earlier age [4]. In contrast, we observed a significantly higher proportion of African American in the “31–40” age subgroup compared to the “18–30” age subgroup (18.0% vs. 12.8%, *P* = 0.008).

Previous studies reported that young NSCLC patients were more likely to have adenocarcinoma histology [2, 4, 6, 7]. Consistent with this, our study showed the “18–30” age subgroup had a significantly higher proportion of adenocarcinoma than the “31–40” age subgroup (66.2% vs. 60.3%, *P* = 0.016). Besides, we also observed a lower proportion of large cell carcinoma in patients under 30 years old (4.7% vs. 8.3%, *P* = 0.008). Young NSCLC patients were reported to have late disease stage at presentation [2, 4, 7]. It was postulated that the lower suspicion of lung cancer in young patients might lead to delayed diagnosis. However, in this study of young NSCLC patients under the age of 40, we found that the younger group had a higher proportion of stage I disease and a lower proportion of stage III disease than the older group. The proportion of stage IV disease was comparable between the two groups. Patients younger than 30 were more likely to receive surgery, This discrepancy in treatment modality may be due to the fact that there were higher proportions of stage I patients and patients with well differentiated tumors while lower proportions of stage III patients and patients with poorly differentiated tumor in the “18–30” age subgroup. Another explanation was that younger patients might be treated more aggressively.

As disease stage was usually the most important prognostic factor, we conducted stage-wise comparison of LCSS between the two age subgroups. Patients under 30 years old had significantly better LCSS in stage I (*P* = 0.038) and stage IV (*P* < 0.001) groups than patients diagnosed at 31–40 years old. Although statistical significance was not reached, there was a trend towards higher LCSS for the younger group with stage II or stage III disease. This result suggested that the association between better LCSS and young patients under 30 was not likely to be confounded by disease stage. Multivariate survival analysis adjusted for race, sex, year of diagnosis, histology, AJCC stage, tumor grade, surgery and radiotherapy also demonstrated the independent role of younger age as a positive prognostic factor.

Molecular mechanisms underlying lung cancer occurring at a young age have been explored in terms of gene polymorphisms [11–13], although there was still insufficient explanation regarding the early onset and distinctive prognosis of this lung cancer subgroup. We hope the results of our study will encourage more research in this field.

In conclusion, young NSCLC patients under 40 years old should not be viewed as a homogeneous entity. Adult NSCLC patients under 30 years old had distinctive clinicopathologic characteristics and cancer-specific survival compared to patients diagnosed at 31–40 years old.

## MATERIALS AND METHODS

This retrospective study was based on publicly available SEER database. Data were retrieved through online access using the SEER*Stat software Version 8.2.1 (http://seer.cancer.gov/seerstat). Informed consent of this study was not required. The Institutional Review Board of Shanghai Chest Hospital approved this study.

### Data collection

We used SEER database 1988–2012 that was submitted in November 2014. To estimate the proportion of patients in the “18–30” and “31–40” age subgroups among all lung cancer patients, we used site codes C34.0-C-34.9 to identify all primary lung cancer patients with ages above 18. The comparison of clinicopathologic features and survival outcomes between the two young age subgroups were limited to patients with NSCLC. Appropriate SEER histology codes ranging from 8010 to 8576 were used to select possible non-small cell lung cancer histologies. Patients without pathologic confirmation of NSCLC, American Joint Committee on Cancer (AJCC) staging information, or sufficient survival data were excluded. Individual data retrieved for each case included age at diagnosis, gender, race, year of diagnosis, tumor histology, tumor grade, AJCC staging information, treatment modality (radiotherapy/surgery/lymph nodes removed/lymph nodes positive), cause-specific death classification, vital status and survival months. Lung cancer-specific survival (LCSS) was defined as time from diagnosis to death from lung cancer.

### Statistical analysis

In this study, young NSCLC patients were stratified into two (18–30 and 31–40) or four (18–25, 26–30, 31–35 and 36–40) subgroups according to age at diagnosis. Pearson's χ^2^ test or Fisher's exact test was used to compare the clinicopathologic characteristics in different age subgroups. Kaplan-Meier method with log-rank tests were used for survival comparisons in univariate analysis. Multivariate survival analysis was performed using the Cox proportional hazards regression to identify independent prognostic factors. Hazard ratio (HR) and its 95% confidence interval (CI) were calculated. Statistical analysis was conducted in SPSS 16.0 (SPSS Inc, Chicago, IL). The yearly bar graph and the line chart showing the proportions of patients in the “18–30” and “31–40” age subgroups among all lung cancer patients were made by Excel. All tests were two tailed, and *P* < 0.05 was considered as statistically significant.
